# Supplementary material for: Are carbon emissions trading and green financial instruments synergistic? -Comprehensive quantitative research based on content analysis
Source: PLoS One. 2024 Mar 7;19(3):e0298601. doi: 10.1371/journal.pone.0298601 (PMC10919601; doi:10.1371/journal.pone.0298601)
Supplement: S1 Data — (ZIP) [file pone.0298601.s001.zip › Data/地级市Z_F工作报告.zip!/║╙─╧/┬σ╤⌠╩╨/└┤╫╘╣1⁄2╓┌║┼í╛┬φ┐╦╩2╛▌═°í┐_2015.docx]

各位代表：

现在，我代表市人民政府向大会作工作报告，请予审议，并请各位政协委员和列席人士提出意见。

一、2014年工作回顾

2014年是本届政府的开局之年。在省委、省政府和市委的坚强领导下，在市人大、市政协的监督支持下，市政府团结带领全市人民，以党的十八大和十八届三中、四中全会精神为指导，紧紧围绕加快建设名副其实中原经济区副中心城市的总体目标，积极适应经济发展新常态，坚持“两提两增四抓”，在极为困难的形势下较好地完成了市第十四届人大一次会议确定的目标任务。

——经济发展呈现好态势。在全国、全省经济增速整体放缓的新常态下，我市经济扭转了2008年国际金融危机爆发以来下行的态势，实现逆势回升。全年实现地区生产总值3284.6亿元，比上年增长9%，增速提高1.8个百分点；固定资产投资2981.1亿元，增长18.3%；社会消费品零售总额1416.3亿元，增长12.6%；外贸出口14.4亿美元，增长11.2%；公共财政预算收入260.3亿元，增长11.2%。

——重大项目实现新突破。323个省、市重点项目完成投资868.2亿元，是年度目标的1.4倍。特别是一批谋划多年而没有干成的项目取得突破性进展：洛阳石化1800万吨炼油扩能改造项目获准开工，一个超千亿级的石化产业正呼之欲出；洛阳城市轨道交通项目获得文物、环保批复，即将取得国家核准；洛吉黄河大桥开工建设，黄河天堑又一条通途指日可待；引黄入洛工程主洞即将贯通，故县水库引水工程顺利开工，更优质的水资源将造福洛阳千家万户；老城历史文化街区项目正式开工，隋唐城“一区一轴”九洲池、应天门项目加快推进，古都厚重的历史文化特色将进一步彰显。

——先行先试带来新红利。我市先后入选国家新型城镇化综合试点城市、信息惠民试点城市、电子商务示范城市、信息消费试点城市、宽带中国示范城市、电子功能材料区域集聚发展试点、首批智慧旅游试点城市、公共文化服务体系示范城市、首批养老服务业综合改革试点城市、中央直属企业“三供一业”分离移交试点城市等，不仅为当前的发展带来了政策资金支持，而且为长远的发展赢得了主动。

——资源挖掘出现新亮点。工业、科技、文化、山水、生态等方面的资源优势正在加速转化为发展优势。机器人及智能装备制造、电子信息等新兴产业扬帆启航，传统制造业借智能化之力加速提升；中油一建研制的世界最大单门吊装系统，中信重工研制的世界最大功率、技术最先进的自磨机，河柴重工研制的国内最大功率高速柴油机，轴研科技获准路试的高铁车辆轴承，让“洛阳创造”享誉海内外；“双申遗”成功再添世界级名片，大遗址保护展示工作成绩斐然，中央新影华夏文化产业园区、白云山国家旅游度假区等一批大项目开工建设，文化旅游业进入新一轮黄金发展期；华夏文明第一河、伊洛河水生态文明示范区等项目顺利推进，林业生态屏障日益完善，魅力独具的宜居之城未来可期。

——载体建设取得新成效。城乡一体化示范区规模以上工业增加值、固定资产投资、公共财政预算收入等主要经济指标增速明显高于全市平均水平，对全市经济的贡献显著提高。产业集聚区进入提质转型创新发展新阶段，固定资产投资突破1500亿元，配套服务和集聚能力明显提升。商务中心区和特色商业区建设全面铺开，泉舜财富购物中心等大型商业综合体建成投用。创新载体加快建设，洛阳大学科技园升格为国家级大学科技园，中意科技园开园运营，浙大科技园、莱普生智慧工场暨物联网创新科技园正式开工。都市生态农业风头正劲，特色农业示范园区“百花齐放”，形成了“一圈四带”的发展格局。

——人民生活有了新提高。预计全年城镇居民人均可支配收入和农民人均纯收入分别达到27054元和9763元，增长9%和11.5%。1.98万户农村危房得到改造。2.3万户城镇居民入住安置房。6520户城市低收入家庭享受到保障房待遇。70852人实现脱贫。城镇居民大病补充医疗保险最高支付额从16万元提高到25万元，异地就医结算城市扩大到20个，城镇居民医疗保险和新农合参保率分别达到98%和99.6%。

——干群精神有了新提振。广大干部在党的群众路线教育实践活动中得到锤炼，作风明显转变，效能不断提升。全市上下心往一处想、劲往一处使，创新求变、干事创业、奋勇争先的氛围日益浓厚、热情空前高涨，已经成为推动洛阳科学发展、跨越发展的强大精神动力。

一年来，我们主要做了以下工作：

（一）面对不断增大的经济下行压力，突出三大举措，加强运行调控，在新常态下实现了经济逆势增长。开放招商增活力。实施十大重点产业招商行动计划，全年共签约亿元以上项目359个，实际利用外资24亿美元。成功引进境内外500强企业12家，正威有色科技城、伊川汽车产业园、阿帕奇电动汽车产业园等一批投资大、带动力强的好项目相继落户。中信重工、一拖集团等一批企业与境外企业合作迈出新步伐，北企集团、北玻公司等一批骨干企业积极拓展海外市场，入选中国外贸出口先导指数样本企业。服务企业解难题。坚持领导分包、落实帮扶政策、搞好要素保障、推进产销对接，强力实施工业“稳增长、调结构、减负担、增效益”百日攻坚行动，支持优势企业扩能增产，帮助困难企业摆脱困境。深入开展银政、银企合作，通过组织银企洽谈会共签约项目5229个、资金793.4亿元，当年落实率达98%以上。大力开展还贷周转金、增信基金、信用评级、中小微企业信贷风险补偿等金融创新服务，缓解了企业、尤其是中小微企业的资金难题。全年金融机构新增贷款334亿元；新增存贷比达到84.7%，创17年来新高。优化环境提效能。深入开展“庸懒散拖”专项治理、百姓问政、群众评诺等活动，行政效能进一步提高。加大企业投诉案件查办力度，企业反映问题解决率达97.1%。加强企业周边环境治理，依法从严打击阻工扰工等违法犯罪行为，保障了企业生产和重点项目建设秩序。

（二）针对结构不优的突出矛盾，启动创新引擎，强化项目支撑，加快产业转型升级步伐。建设平台推动科技创新。出台促进产学研结合十条新政，支持企业开展产学研协同创新，新增院士工作站6家、市级以上研发平台83家、高新技术企业孵化器4个，40家企业入围高新技术企业和创新型试点企业。先后与中科院自动化所、计算所、微生物所和上海交通大学、华大基因合作在洛组建创新机构。荣获国家科技进步奖1项、国家技术发明奖3项、河南省科技进步奖35项。专利申请量、技术合同成交额同比分别增长17.5%和29.5%。高新区被确定为全省唯一的国家知识产权示范园区。扩大投资推动工业结构调整。实施重大工业结构调整项目420个，带动全市完成工业投资1411.5亿元。围绕延链补链，启动有色金属、石化煤化等重点产业转型升级计划，中建材新型显示材料基地、恒阳铝业高档铝型材等162个项目开工建设；中信重工重装高科产业园、华茂新材料产业园等258个在建项目顺利推进；河南能源20万吨乙二醇、洛阳单晶硅半导体产业园等97个项目竣工投产。实施电子信息、智能装备制造等产业培育计划，以“一基地两园区”和30家骨干企业为重点，着力培育机器人及智能装备产业集群。引进培育电子信息企业150家，惠普国际软件人才及产业基地、中兴通讯智慧产业基地等一批重大项目开工建设，中航锂电锂离子动力电池二期、中航光电技术产业基地等项目竣工投产。21个重大环保技术装备项目列入国家鼓励发展目录，数量居全省首位。转变方式推动现代农业健康发展。实施农业产业化集群和龙头企业培育工程，新增市级以上龙头企业86家、专业合作社148家，农业产业化占农业总产值的比重达60%；积极发展适度规模经营，培育新型农业经营主体，新增家庭农场106个，完成土地流转101万亩。成功创建出口牡丹芍药花卉国家级质量安全示范区。洛建农业成为全省首家在上海股交中心E板上市的农业企业。多策并举推动现代服务业快速发展。出台《关于加快发展高成长服务业的实施意见》，确定八大领域、六大园区的总体发展战略，中国移动呼叫中心一期主体工程完工，五洲国际工业博览城、八里·唐文化创意产业园等项目加快实施。哈他网络公司入选全省电子商务示范企业。第32届中国洛阳牡丹文化节成功举办。全省首家智慧旅游指挥中心、城市旅游集散中心顺利投用。全年接待游客9470万人次，旅游总收入突破600亿元、增速高于全国9.3个百分点。

（三）针对影响发展的体制机制障碍，全面深化改革，实施重点突破，加速释放发展活力。正式启动新型城镇化试点。编制完成试点实施方案，出台户籍制度改革等相关配套政策，扎实开展市民化体制机制改革、农村集体土地改革、农村土地承包经营权确权登记颁证等试点工作。稳步推进新一轮国企改革。出台深化国资国企改革意见和实施计划，积极推动洛粮公司、有色矿业等通过改制重组发展混合所有制经济。深化与央企、省企战略合作，启动32家央企“三供一业”分离移交工作。顺利完成白马集团纺织搬迁升级改造。多点突破推进投融资制度改革。引进北大方正等社会资本参与医疗卫生、交通、能源、市政等基础设施领域建设经营。组建宏科创新创业公司，搭建机器人及智能装备制造产业投资平台。顺利争取到财政部和亚洲开发银行PPP模式试点城市。成功发行洛阳银行50亿小微企业金融债、洛钼股份49亿元可转债和中信重工10亿元公司债。全省首家金融租赁公司和我市首家外商投资融资租赁公司开业运营。8家企业成功在“新三板”挂牌，通达电缆、轴研科技在资本市场再融资7.2亿元。深入推动政府机构改革。调整理顺工商、质监、食品药品监管行政管理体制，减少市政府工作部门4个。稳步推进县（市、区）政府机构改革。进一步完善事业单位绩效工资制度。继续深化行政审批制度改革。积极推进行政审批“两集中、两到位”，加快网上审批试点工作，探索建立行政部门权力清单，取消市级行政审批事项74项、下放37项。出台企业投资负面清单。深入推进工商登记制度改革，新增企业户数、新增企业资本总额同比分别增长72.5%和198%。全力推进非公有制经济健康发展。大力实施“小巨人”培育工程和“百千万成长工程”，非公有制经济实现增加值同比增长13.3%，对全市经济的贡献率达到57%。

（四）遵循新型城镇化的发展要求，突出示范引领，完善基础功能，持续改善城乡面貌。增强城乡一体化示范区引领作用。示范区第二污水处理厂、中原大道等项目启动建设，华能热电联产二期工程第一台机组即将投运，高铁大道、希望路跨伊河大桥等一批重大基础设施项目竣工投用。智慧旅游、智慧教育、智慧园区、智慧楼宇等四大智慧城市样板点部分建成投用。持续提升城市支撑能力。加紧实施东、西、南、北环城道路，连霍高速洛阳至三门峡段改扩建工程竣工通车。新建、改扩建城市道路26.7公里、高速公路28.9公里、县乡公路238公里。开工建设城市生活垃圾综合处理园区、华润首阳山电厂至洛阳市区长输供热管网。新增供热面积300万平方米。西工、伊新南等6座变电站建成投用。3G网络覆盖城区，4G网络覆盖热点区域。数字化城市管理系统高效运行。加快棚户区改造和县城建设。坚持安置优先，稳步推进旧城棚户区城中村改造，全年累计开工1884万平方米、竣工1788万平方米。积极推进县城提质扩容和重点镇、旅游名镇建设。扎实推进美丽乡村建设。885个行政村环境集中整治全部达标，81个美丽乡村试点建设顺利实施。硬化通组通户道路2013公里。解决农村饮水安全46.1万人。实施产业化扶贫项目269个，完成扶贫搬迁4.4万人，社会化扶贫工作受到国家表彰。老区建设取得新成效。我市被确定为全省改善农村人居环境示范市。加强生态环境系统建设。积极创建国家环保模范城市，5个重点企业资源综合利用项目、5个循环经济园区“城市矿产”示范基地项目竣工投用，14个综合节能改造项目进展顺利，全年单位地区生产总值能耗下降5.5%左右。137家工业企业实施废气治理，80项重点减排工程、27台电力机组除尘脱硫脱硝设施建成投用，拆除燃煤锅炉32台，淘汰黄标车、老旧车2.9万辆。大气质量、主要河道断面水质达到省定目标。完成造林45.3万亩、林业生态圈建设10.8万亩；市区新增绿地214.7公顷，城市绿化覆盖率达39.6%。栾川县入选“国家园林县城”，被确定为全省唯一的国家生态文明建设示范区。

（五）顺应全市人民的美好期待，倾力改善民生，促进社会和谐，不断提升群众幸福指数。持续加大民生投入，全年各级财政用于民生的支出达296.1亿元，占公共预算支出的71.9%。省定民生工程、市定民生实事全部完成。促进就业创业。强化技能培训和就业帮扶，新增城镇就业11.5万人、再就业3.5万人。在全省率先开展灵活就业人员助保贷款工作，发放小额担保贷款17.6亿元，扶持2.7万人成功创业。全年培训农民10.3万人次。新增农村劳动力转移就业3.7万人。完善社会保障体系。在全省率先实现县乡村三级公立医疗机构药品零差率销售全覆盖。全市二级以上医疗机构和乡镇卫生院全面实行“先看病、后付费”模式。启动实施新农合大病医疗保险。加快市第五人民医院新区医院、河科大一附院新区医院二期等工程建设，市妇幼保健院新区医院建成开业。开工保障性住房2.7万套、竣工1.9万套。启动县级社会福利中心建设，新建农村老年幸福院284所，新增养老床位3200张。全面发展社会事业。新建、改扩建中小学、幼儿园64所，引进名牌学校6所。启动集中连片特殊困难地区普通高中改造。美丽园丁教育基金培训项目累计培训教师1.59万人次。洛阳师范学院新校区和职教园区4所职业学校二期项目全面启动。我市顺利通过国家语言文字二类城市评估。扎实推进国家公共文化服务体系示范区创建工作，新编戏剧《穆桂英挂帅》、《洛阳令》广受好评。组织文化惠民演出600余场。孟津、新安荣获“全国文化先进县”称号。新增全民健身活动场地50余万平方米。我市荣获全国体育事业突出贡献奖和第12届省运会金牌、奖牌、总分一等奖。不断创新社会管理。110联动工作实现全覆盖，群众诉求办结率和满意率均达95%以上。启动实施单独二孩政策。加强安全生产监管，深入开展食品药品安全专项整治，积极化解信访积案，提高社区网格化管理水平，依法打击处置非法集资，严厉打击违法犯罪活动，社会大局保持稳定。

（六）以党的群众路线教育实践活动为载体，坚持依法行政，大力改进作风，持续加强政府自身建设。扎实开展群众路线教育实践活动。以“为民务实清廉”为主题，深入开展“五查五看五争当”活动，针对查摆出来的“四风”突出问题，认真制定整改措施，着力解决好政策落实“最先一公里”、联系服务群众“最后一公里”问题，作风建设的常态化环境、长效化机制进一步形成。主动接受人大及其常委会监督，积极支持政协参政议政，“制订中原经济区副中心城市建设规划”、“提高教育均衡化发展水平”等议案得到较好落实，人大代表建议和政协提案办理效果良好。提请市人大常委会审议地方性法规草案3部，出台政府规章1件，清理规范性文件1788件。强力推进政府廉政建设。严格执行公务消费、用车、住房管理制度，加大审计监督力度，“三公经费”同比下降28.8%。坚持以零容忍的态度惩治腐败，共查办案件2154件，给予党政纪处分2119人次；查处损害群众利益问题284件，追究责任241人次。与此同时，军民融合发展质量和国防动员保障能力进一步提升，双拥工作取得新成效。民族宗教、外事侨务、对台、统计、审计、人防、气象、地震、档案、史志、残疾人工作、妇女儿童事业等都取得了新的成绩。

各位代表！过去的一年，不平凡、不容易、不简单！回顾一年来的工作，尽管困难很多，但是结果超出预期。我们深切体会到：洛阳之所以能够在巨大的困难面前破浪前行、砥砺奋进，正是得益于省委、省政府和市委的坚强领导，得益于历届领导班子和老同志打下的坚实基础，得益于市人大、市政协、各民主党派、人民团体的监督支持，得益于全市人民和社会各界的团结奋斗、无私奉献。在此，我代表市人民政府，向辛勤工作在各条战线的全市人民，向人大代表、政协委员，向各民主党派、工商联、人民团体，向离退休老领导、老同志，向驻洛部队、武警官兵和公安干警，向所有关心支持洛阳经济社会发展的社会各界和海内外朋友，表示衷心的感谢并致以崇高的敬意！

在肯定成绩的同时，我们也清醒地认识到，我市经济社会发展中仍存在一些突出问题：经济回升向好的基础还不稳固，结构性矛盾尚未得到有效解决；各种资源优势尚未得到充分发挥，城市综合竞争力和辐射带动能力还不够强；城乡二元结构矛盾依然突出，农民增收、农村脱贫的难度增大；安全生产、环境保护、食品药品安全等领域还存在薄弱环节；公共服务体系还不完善，就业、社保、教育、医疗、住房等群众关心的问题没有得到妥善解决；政府职能转变还不到位，一些机关工作人员法治意识、服务意识、担当意识不强，行政效率不高，一些部门和领域腐败现象还时有发生。对于以上问题，我们一定高度重视，认真解决，决不辜负全市人民的期望和重托！

二、2015年总体要求及重点工作安排

今年是全面深化改革的关键之年，也是“十二五”规划的收官之年，更是洛阳站在新的发展起点、全面实施中原经济区副中心城市战略的开局之年。

站在新起点，认识新常态，洛阳加快发展的政策利好很多、机遇十分难得！我国经济发展进入新常态，经济总体向好的基本面没有改变，洛阳发展仍然面临着较好的宏观条件和稳定的政策环境。东部沿海地区产业加快向中西部转移，新一轮科技革命和产业变革催生了一批新技术、新业态、新模式，“一带一路”重大经济战略加快实施，一系列重大改革将密集面世，一大批推动结构调整、促进创业创新、扩大消费升级的重要举措将陆续出台。特别是我市被列为国家新型城镇化和信息惠民等试点城市，加之洛深合作全面启动，将在扩大有效投资、承接产业转移、提升创新能力、加强城乡建设等方面为我们注入新的活力。只要我们主动适应新常态，牢牢把握新机遇，就一定能够抢占先机，赢得主动，打造洛阳经济的“升级版”！

站在新起点，明确新定位，洛阳未来发展的蓝图已经绘就、路径更加清晰！中央加快转变经济发展方式、促进提质增效的态度非常坚决，宏观调控的政策导向十分明确。省委、省政府确定的“三大国家战略”加快实施，“四个河南”建设方兴未艾。市委十届十三次全会描绘了洛阳加快建设中原经济区副中心城市的美好蓝图，进一步明确了中原经济区重要增长极、文化示范区、最佳宜居地、开放创新城的战略定位。只要我们咬定青山不放松，坚持与省会郑州错位发展，紧盯副中心城市的目标定位持续发力、久久为功，就一定能够把美好的愿景变成现实！

站在新起点，实现新跨越，洛阳加快发展的能量正在蓄积、活力前所未有！改革开放30多年的持续快速发展，特别是近几年，一大批打基础、管长远、增后劲的重大基础设施项目和产业项目顺利实施，为洛阳下一步的发展打下了坚实基础；近年来应对危机的挑战让我们磨炼了意志、积累了经验；日趋激烈的区域竞争使我们时刻保持清醒和忧患，做好了竞赛和拼搏的充分准备。只要我们团结一心、和衷共济，就一定能够凝聚起加快发展的强大力量！

站在新起点，面对新期待，洛阳人民对美好生活的向往更加迫切、我们使命光荣！解决好老百姓关心的民生问题，让人民生活得更加幸福，是人民政府应有的担当。随着经济社会的快速发展，人民群众的需求更趋多元，更加注重教育、医疗、住房、环境等方面品质的提升。同时，越来越多的洛阳人，在城乡建设、民生改善中得到了实惠，看到了洛阳发展的希望。只要我们想群众之所想、急群众之所急，在增进人民福祉上多谋事、在破解民生难题上干实事，就一定能够赢得全市人民的拥护和支持！

2015年，政府工作的总体要求是：深入贯彻党的十八大和十八届三中、四中全会以及中央经济工作会议精神，认真落实省委九届八次全会和省委、市委经济工作会议精神，紧紧围绕建设名副其实的中原经济区副中心城市，坚持调中求进、改中激活、转中促好、变中取胜，统筹推进稳增长、调结构、促改革、惠民生、创优势、防风险等工作，运用互联网思维，全力推动大众创业、万众创新，探索走好新常态下全面协调可持续发展之路，努力保持经济平稳增长、社会和谐稳定。

初步确定2015年全市经济社会发展的预期目标是：地区生产总值增长9%以上；地方公共财政预算收入增长10.5%；固定资产投资增长16%；社会消费品零售总额增长13%；外贸出口增长10%；居民消费价格指数控制在3%以内；城乡居民收入增长9%以上；节能减排完成省下达任务。

各位代表！实现上述目标，关键是要坚定不移地沿着市委十届十三次全会确定的《建设中原经济区副中心城市战略纲要》路线图，深化开放招商、服务企业、优化环境等重要举措，抓好产业升级、深化改革、创新驱动、文化复兴、生态建设、城乡统筹、改善民生等重点工作，进一步强化组织保障、法治保障、人才保障、资金保障、作风保障、舆论保障，加快建设中原经济区的重要增长极、文化示范区、最佳宜居地、开放创新城，统筹做好以下几方面的重点工作：

（一）坚持工业转型发展、融合发展，构筑中原经济区重要增长极的战略支撑。推动优势产业延链补链、战略新兴产业集聚发展，实现工业结构向产业链和价值链高端转变，加快建设先进制造业大市。全年安排新开工亿元以上工业结构调整项目340个，计划投资650亿元以上。以推动传统优势产业高端化为目标，大力实施机器人及智能装备产业攻坚行动计划，带动轴承、齿轮、控制系统等智能装备产业全面发展，推动“洛阳制造”向“洛阳智造”转型；实施企业生产设备数字化改造，培育一批数字化车间、智能化工厂；抓好有色金属产业的延链和补链，积极开发轨道交通装备产业需要的铝合金，精密仪器、军工等产业需要的钨钼硬质合金，电力、电子产业需要的铜合金等应用领域产品；推进石油化工与新型煤化工融合发展，大力实施洛阳石化炼油扩能“一主四辅”工程，扩大乙二醇现有产能；加快煤制氢、PTA、环氧丙烷等项目前期工作，力争尽快开工建设；以创建国家级军民结合新型工业化产业示范基地为契机，推进军品和民品融合发展，重点发展一批大型军民结合企业，服务好与军品配套生产的中小企业，拓展完善上下游产业链，带动配套企业数量增加、规模扩张、产值提升。以促进战略新兴产业规模化为引领，大力实施电子信息产业三年行动计划，促进伊川、伊滨等电子信息产业园区集聚发展，半导体材料、芯片、平板显示等产品尽快形成规模产能，构建完备的电子信息产业体系；加快建设新材料国家高技术产业基地，培育高端钨钼钛合金材料、电子功能材料产业集群；大力发展光伏、锂离子动力电池、新能源汽车、风电关键部件和装备；推动现代中药、化学合成药以及兽用疫苗、诊断试剂、兽药等产业快速发展。以加快园区特色产业集群化为导向，实施产业集群龙头企业、优势产业集群培育工程，尽快形成装备制造、有色金属、石化煤化等3个千亿级产业集群和一批超百亿特色产业集群；理顺产业集聚区体制机制，实现集聚区与所在乡镇机构套合设置；创新融资模式，加快生产配套、生活服务、创业创新平台建设，完善公共服务功能，提升集聚区吸纳能力。

（二）坚持调整农业结构、转变发展方式，夯实中原经济区重要增长极的坚实基础。适应“生产导向”向“消费导向”转变的要求，走出一条农业产业化和现代化的新路子，让农业更强、农民更富、农村更美。持续优化农业结构。依靠科技支撑，把产业链、价值链等现代产业组织方式引入农业，完善种、养、加、销全产业链，实现现代农业接“二”连“三”。积极发展优质粮油及核桃、烟叶、中药材等经济作物和林果业，鼓励支持农产品精深加工，建设一批规模化、标准化种养基地，培育壮大农业特色产业、支柱产业。健全农业科技机构和人员激励政策，加强农业科技攻关。深化与华大基因的战略合作，加快牡丹、谷子等新品种、新科技研发推广。大力发展都市生态农业和休闲农业。以绿色生产、高质高效、观光休闲为主，建设5—10个市级都市生态农业示范区，新增休闲农业园区15家。积极发展生态循环农业，推广农业标准化生产，加快农产品出口质量安全示范区建设，争取再有2家企业入选省级以上示范区。加快构建新型农业经营体系。鼓励适度规模经营，新发展市级农业产业化龙头企业20家、农业产业化集群5个、农民合作社和家庭农场各50个。完善农业保障服务体系。充分发挥政策惠农效应，千方百计促进农民增收。加快小型水库、塘堰坝除险加固及抗旱吃水工程建设，确保前坪水库年内开工建设，小浪底南岸灌区等重大项目前期工作实现突破。完成中小河流治理项目10个，治理水土流失150平方公里，增加有效灌溉面积6万亩。新增农机动力5万千瓦以上。扩大农业政策性保险覆盖面，减少灾害损失。支持电商、物流、商贸、金融等企业参与涉农电子商务平台建设，拓展网络销售平台，打造洛阳特色农产品品牌。用好农民大学培训平台，加强农民职业技能培训。加快建设美丽乡村。持续抓好美丽乡村试点建设，重视偏远乡村环境综合整治，完成1015个环境集中整治达标村创建任务。改造农村危房5000户。硬化通组通户道路2000公里。完成饮水安全任务，让所有农村居民都能喝上干净的水。建立留守老人、儿童、妇女关爱服务体系。加大乡土文化及传统村落保护力度，让人们望得见青山、看得见绿水、记得住乡愁。

（三）坚持服务业扩容提升、多点支撑，打造中原经济区重要增长极的新引擎。新常态下，社会消费加速从生存型消费转向享受型、发展型消费。要挖掘历史文化、山水生态、中医中药等资源优势，大力发展高成长性服务业，加快培育新的经济增长点。促进文化旅游业深度融合发展。以建设中原经济区文化示范区为目标，大力推动“文化复兴”，充分利用和活化历史文化资源，加快隋唐洛阳城“一区一轴”、汉魏洛阳城等大遗址保护展示工程建设，积极开展二里头夏都文化遗址博物馆建设前期工作，规划建设丝绸之路博物馆、契约博物馆、仓窖博物馆，叫响“世界圣城、丝路起点、千年帝都、牡丹花城”四大城市文化名片。支持文化创意、影视基地、数字出版、动漫游戏等文化产业发展，尽快形成一批文化产业集聚区。加快旅游干线公路、景区间互通道路等基础设施建设，重点抓好中央新影华夏文化产业园区、白云山国家旅游度假区等项目，推动旅游与文化融合发展，促进伏牛山休闲度假游带、沿黄河休闲度假游带提档升级，形成休闲度假高端文化旅游品牌。以智慧旅游城市试点为载体，加快公共服务、综合监管、宣传营销等平台建设，提升旅游服务水平。全年接待游客突破1亿人次，实现旅游总收入700亿元以上。优先发展现代物流业。依托现代综合交通网络，进一步完善设施、提升功能，培育电子、家电、建材、农副产品等一批专业物流基地。推动电子商务与物流业融合发展，提升物流配送能力，打造区域性快递分拨中心。加速扩张信息服务业。加快信息服务业园区建设，积极培育物联网、大数据、云计算、移动多媒体等新型业态。加快惠普国际软件人才及产业基地、中兴通讯智慧产业基地等重大项目建设，打造智慧城市的新样板和智慧产业的新高地。以建设国家电子商务示范城市为载体，推动电子商务产业集群发展，着力打造区域性电商产业基地。大力发展服务外包产业，争创国家服务外包示范城市。培育壮大科技服务业。依托各类科技创新载体，推动技术集成创新，积极发展新型科技服务业态，重点抓好研发设计、创业孵化、科技咨询、科学技术普及等科技服务。大力发展股权质押、知识产权质押、信用保险质押等科技金融产品和服务。积极发展金融服务业。支持洛阳银行跨区域发展，促进金融租赁公司健康运营。全面推进县级农信社改制组建农商行工作。支持保险机构开展贷款保证保险、贸易信用保险，扩大保险产品融资规模。大力引进外资银行、股份制银行及非银行金融机构在洛设立分支机构。积极发展互联网金融。提升发展商贸服务业。以“两区”建设为重点，引领城市商业业态转型升级，尽快形成产业高集聚、功能高复合、就业高容量的商贸服务业集群。抓好城市综合体、现代专业市场建设，加快发展楼宇经济，增强商务商贸集聚功能。不断壮大健康服务业。发挥生态、医疗、中药等资源优势，推动文化、旅游、医药、社区等业态融合发展。坚持政府引导、市场驱动，谋划建设一批复合型健康养老项目，打造健康养老产业集群。大力发展会展业。利用牡丹文化节等平台，整合资源，丰富内涵，积极申办、引进知名展会、商务会议，培育特色展会品牌。引进专业公司，提高会展市场化运作水平。稳步发展房地产业。落实促进房地产市场平稳健康发展的政策措施，优化房地产业结构，因地制宜发展商业地产、旅游地产等，推动房地产业走出低谷。

（四）坚持开放带动、创新驱动，打造中原经济区的开放创新城。持续深化开放招商，全面加大创新力度，增强经济发展后劲，培育新的增长动力和竞争优势。强力推进重点产业招商。由靠优惠政策招商向亲情招商、以商招商转变，向优化环境、真诚服务转变，完善客商投资项目审批代理制，搞好项目前期、中期和后期服务，确保项目引得来、能落地、快开工、早投产。按照“大项目—产业链—产业集群—产业基地”的思路，细化十大重点产业招商路线图，紧盯优势企业积极对接，加快引进一批龙头型、基地型项目，吸引配套企业跟进，提高主导产业集聚度和竞争力。加强与有意来洛投资、特别是在谈的战略投资者的对接，力争在汽车及零配件、高端轴承、通用航空、新能源汽车、现代物流、健康养老等领域实现突破。完善“四个一”工作机制，抓好签约项目的跟踪落实，力争当年招商项目履约率达到95%以上，开工率达到70%以上。积极融入“一带一路”规划。谋划建设丝绸之路文化交流中心。深化与意大利都灵市在新型城镇化、节能环保、新能源等方面的合作，加快中意科技园建设。支持中储公司建设保税物流中心，完善铁路集装箱站口岸功能。优化出口结构，加强摩托车、太阳能光伏、钢制办公家具、新材料和装备制造等优势产品出口基地建设，培育壮大龙头企业，打造洛阳出口品牌。积极组织参加广交会、亚欧博览会等，拓展俄罗斯、中东、印度等新兴市场，不断扩大出口规模。支持企业“走出去”，参与海外资源开发和国际工程建设。推动创新能力加速提升。集聚技术、人才、资本等创新要素，有效整合资源，集成落实政策，完善服务模式，培育创新文化，营造万众创新的社会氛围。围绕机器人及智能装备、新材料、电子信息等重点产业部署创新链，落实产学研重点行动计划。加快中科院、北京航空航天大学、上海交通大学等知名科研院所、高校在洛研发机构建设，新建市级以上研发中心60个，力争突破一批产业关键核心技术，加快科技成果的产业化步伐。全年专利申请量突破1万件。强化人才智力支持。完善人才培养引进机制，统筹运用产业配套、政策优惠、资金扶持、环境优化、情感关怀等综合性措施，加快集聚一批实用型、技能型特别是领军型优秀人才和创新团队，打造令人向往的人才高地。

（五）坚持深化改革、创新体制，最大限度地激发经济活力。按照中央和省里部署，有序推进重点领域改革，努力把改革的新红利转化为发展的新动力。持续推进国资国企改革。促进市属国有企业股权多元化，大力发展混合所有制经济，建立健全现代企业制度；实施国有企业分类监管和以管资本为主的新型国资监管；加快政府投融资公司及国有资产整合步伐；深化与央企省企战略合作，支持其利用增量资产或剥离部分生产经营职能，与地方企业重组。驻洛央企“三供一业”分离移交工作今年要实现大头落地。建立市场化的投融资机制。大力推进投融资模式创新，积极探索政府与社会资本合作模式，吸引民间资本参与城市基础设施和教育、卫生等公共服务项目建设，增强公共产品供给能力。鼓励企业通过IPO、“新三板”拓宽股权融资渠道，通过发行企业债、中期票据、私募债扩大直接融资规模。切实抓好重点领域改革。统筹推进医疗保障、公共卫生、药品供应、监管体制等综合改革。积极稳妥推进机关事业单位养老保险制度改革。基本完成党政机关公务用车改革。深化交通运输综合执法、交通管理体制及市公交集团改革。稳妥推进资源性、公共性产品和服务价格的市场化改革。加快财政体制改革，完善预算管理制度，防范政府债务风险。全面深化农村改革，抓好土地承包经营权确权登记颁证工作，建立农村产权交易中心，引导土地经营权规范有序流转。推进农村集体经济股份制改革，激发农村经济活力。深化文化、教育、环保等领域的改革。大力发展非公有制经济。设立政府创业投资引导基金，打造网络化、开放式的创业服务平台，推动大众创业、万众创新。大力实施“小巨人”企业培育工程，加大金融对实体经济和中小微企业的支持力度，帮助“草根经济”长成“参天大树”。进一步清除市场准入障碍和各种隐性壁垒，支持非公有制企业平等参与基础设施和民生领域建设、参与国有企业改革和事业单位改制重组。

（六）坚持城乡统筹、服务延伸，构建城乡一体化发展的新格局。突出新型城镇化龙头带动作用，以规划引领优化空间，推动城乡统筹建设、产业协调布局、公共服务均等化发展。加快推进新型城镇化试点工作。主动先行先试，积极探索农业转移人口市民化成本分担、多元化可持续投融资等体制机制，坚持“一基本两牵动三保障”，积极引导农民进城，让农业转移人口进得来、留得住。增强中心城区承载辐射能力。谋划建设城市快速公交和快速路“双快系统”，积极推进新街跨洛河大桥、孙辛路高架、九都路东延长线、东环城路等重点工程，打通郑州路、道北三路等7条断头路，缓解城市拥堵压力。加大棚户区改造力度，全年实施棚户区（城中村）改造项目200个，安置群众2.2万户。以中心城区为核心，辐射带动周边卫星城，引导招商项目和城区大工业配套项目向县域合理布局，增强县域承载能力和配套功能，持续推进重点镇、中心镇、特色景观旅游名镇建设，促进中心城市和县城、小城镇合理分工、功能互补、协同发展，努力形成独具洛阳特色的城市群。强化城乡一体化示范区带动作用。加快万安山综合开发区、高铁龙门站周边区域、隋唐城遗址里坊区等重点片区的规划建设，在人口集聚转移、城乡社会管理一体化等方面先行先试，推进示范区全域公交同城、环境同治、基础设施协调同建、公共服务均等化。以伊滨产业集聚区、经济技术开发区、洛龙高新技术产业开发区为平台，着力打造高端产业、高端人才的集聚地。开工建设环城南路、滨河南路等骨干道路，加快中原大道、伊滨公园二期、示范区第二污水处理厂等项目建设。提高城乡基础设施支撑保障水平。积极构建城市综合交通体系，加快城际铁路、城市轨道交通、武西高速等项目前期工作，争取310国道、洛吉快速通道二期等项目早日竣工。继续推进农村公路改造提升三年行动计划。积极开辟和优化境内外航线，争取开通1—2条国际航线并实现常态化。抓好天然气、煤层气入洛管线及县县通燃气管网建设，形成多气源供气格局。加快故县水库引水、华润首阳山电厂至洛阳市区长输供热管网等项目建设。积极发展热电、光伏发电和高效大机组，加快大鱼沟抽水蓄能电站等项目前期工作，抓好万基2×60万千瓦机组、华能瑞阳2×35万千瓦热电联产以及26个110千伏以上电网项目。出台绿色建筑实施意见，积极推广建筑绿色节能。加快智慧城市建设，实施数字化城市管理系统二期工程，深入开展市容市貌提升和专项整治行动，重点治理店外经营、乱停乱放、露天烧烤等城市顽症，提高精细化管理水平，确保通过国家卫生城市复审。

（七）坚持生态创建、绿色发展，建设中原经济区的最佳宜居地。遵循“绿色低碳环保”理念，加大生态保护力度，构建城乡一体的良好生态系统，让人们看到更多的蓝天白云，喝到更加干净的水，呼吸到更加新鲜的空气。做好“山”文章。围绕白云山、老君山、龙门山、青要山、万安山、周山等市内名山，加强环境整治、矿区生态修复和自然保护区建设，持续强化水土流失治理，打造山地旅游休闲度假养生新亮点。做好“水”文章。谋划实施一批沿河生态带、引水蓄水治水工程，加快建设全国水生态文明城市。抓好伊洛河水生态文明示范区、华夏文明第一河、龙门国家湿地公园等重点项目，合理开发、科学利用小浪底、西霞院等境内水库资源，建设名副其实的北方水城。深入开展城市区“四河七渠”水环境整治，治理河道47公里，确保城市区排污口全部截流；建成产业集聚区和乡镇污水处理厂，加快部分县城第二污水处理厂和村级污水处理设施建设。做好“气”文章。实施水泥、玻璃、陶瓷等行业提标治理，确保全部达标排放。加强工业废气、秸秆焚烧、机动车尾气、建筑施工和道路扬尘治理，持续抓好燃煤锅炉拆改、黄标车淘汰等工作，购置新能源公交车300辆，严控主要污染物排放，推动绿色低碳生产生活。严厉打击环境违法行为，让损害环境者付出惨痛代价。做好“绿”文章。高标准实施宜林荒山绿化和高速、快速公路沿线通道绿化，加快林业生态圈建设，形成“一廊一带三组团”格局，全年造林绿化50万亩以上，全市森林覆盖率达到47.5%。加快城市公园、广场和公共绿地建设，城市绿化覆盖率达到40.5%。

（八）坚持改善民生、创新管理，大力提高人民群众生活质量。更加关注和改善民生，认真办好惠民实事，让洛阳人民的生活一年更比一年好。努力推动高质量创业就业。全面落实就业扶持政策，大幅度增加小额担保贷款基金，放宽审批条件，强力推进大众创业，特别是促进大学生创业就业，构建面向人人的“众创空间”等服务平台，激发群众创造活力，进而带动扩大就业。实施职业教育攻坚计划，完成职业技能培训1万人。全年新增城镇就业10万人、再就业3万人。落实同工同酬政策，建立农民工工资正常支付的长效机制，依法保障农民工劳动报酬权益。新增农村劳动力转移就业3万人。加快构建完善的保障服务体系。加大扩面征缴力度，城乡居民养老保险基础养老金标准每人每月提高18元，企业退休人员基本养老金再提高10%。新农合和城镇居民医保财政补助标准每人每年再提高60元；实现城镇居民医保参保100万人以上。推进异地就医即时结算，推广居民医保门诊统筹即时结算，逐步实现城乡居民就医“一卡通”。加强基层医疗卫生机构建设，指导乡镇卫生院探索开办小型特色专科或康复型养老机构。新建农村老年幸福院200个、新增养老床位2000张以上。开工各类保障房7.3万套、竣工2.4万套。切实办好人民满意的教育。实施学前教育三年行动计划，推动普惠性幼儿园和“公建民营”幼儿园发展。编制完成并实施中小学、幼儿园专项规划，加快市区薄弱学校建设，改善贫困地区办学条件，促进义务教育均衡发展。支持在洛高校发展，加快洛阳职业技术学院筹建步伐。繁荣发展文化体育事业。完善公共文化服务设施网络，加快市文化馆等公共文化设施项目建设，开展农村数字电影、社区公益电影放映和百场公益性文化演出等文化惠民活动，鼓励文艺精品创作，推进全民健身活动蓬勃发展，确保我市创建国家公共文化服务体系示范区工作顺利通过验收。持续加大扶贫攻坚力度。实施精准扶贫、科学扶贫，多方筹措资金加大扶贫投入，坚决打赢扶贫开发攻坚战。积极谋划扶贫项目，实施贫困村整村推进120个、产业项目255个，扶贫搬迁7000户，解决5.95万人脱贫问题。深入推进社会治理创新。加快国家信息惠民试点城市建设，以信息化带动和促进民生改善。畅通群众信访渠道，全面推行重大决策社会稳定风险评估机制，开展依法逐级走访、诉访分离和积案化解工作，妥善解决群众合理诉求，引导群众依法、理性维权。下大力气整顿和规范小额贷款、融资性担保行业，坚决防控金融风险。深入推进平安洛阳建设，突出“双安双治双基”，严密防范、严厉打击各类违法犯罪行为。健全市、县、乡三级食品药品安全监管网络，让群众放心用餐、用药。加强安全隐患排查治理体系建设，持续开展重点行业（领域）专项整治及“打非治违”行动，杜绝重特大事故发生，确保社会和谐稳定。认真做好新形势下的国防动员和民兵预备役工作，争创全国双拥模范城“七连冠”。加强人口与计划生育工作，确保人口自然增长率控制在7.1‰以内。支持工会、共青团、妇联等人民团体工作。做好民族宗教、外事侨务、对台、统计、审计、人防、气象、地震、档案、史志、残疾人事业等工作。

同时，要认真学习深圳等地先进经验，加强重大战略专题研究，积极开展专题调研、专家访谈、市民建言献策等活动，认真编制好“十三五”各项规划。加强政策研究和项目谋划，做好各项基础工作，争取更多的项目进入国家、省规划盘子。

三、全面加强政府自身建设

深入开展“三严三实”主题教育活动，推进作风建设常态化、长效化，全面提高政府工作的科学化、法治化水平，让为民务实清廉的政府形象更加深入民心。

依法行政，建设公平正义的法治环境。完善政府立法，严格文明执法，提高执法效率和规范化水平，让政府权力在法治轨道上规范运行，让群众在法治建设中感受到公平正义。全市各级政府及部门工作人员要主动学法，自觉守法，善于用法，切实提高运用法治思维和法治方式推动政府各项工作的能力。自觉接受人大及其常委会的法律监督、工作监督，主动接受政协的民主监督，诚恳接受新闻媒体和社会公众的监督。完善工作沟通、落实和反馈机制，提升人大代表议案、建议和政协提案办理质量。完善市政府领导联系民主党派、工商联制度，积极推进政府协商民主工作。加大法律援助力度，维护好困难群体的合法权益。建立政府法律顾问制度，依法健全完善公众参与、专家论证、风险评估、合法性审查、集体讨论决定等决策程序，让政府的每一项决策都经得起法治的考量、体现出民意的力量。

转变职能，营造竞争有序的市场环境。扎实推进政府机构改革，全面完成省定改革任务；稳步推进事业单位分类改革，解决好生产经营性事业单位转企改制遗留问题，扩大政府购买社会服务规模。大刀阔斧简政放权，进一步压缩审批事项，全部取消非行政许可审批，探索建立政府行政审批目录清单、政府行政权力清单、政府部门专项资金管理清单和行政事业性收费目录清单，加快企业投资项目核准改革试点工作，用政府的有所不为换来市场主体和社会力量的大有作为。

创新服务，打造国内一流的发展环境。围绕“大服务、真服务、常服务”，创新服务企业理念，坚持生产要素协调与支持服务企业技术创新、产品创新、营销创新、机制创新、市场开拓并重，助推企业调结构促转型、拓市场稳经营、降成本增效益。加强政府信用体系建设，认真履行对企业的承诺。以沟通企业“零距离”、服务企业“零障碍”、损害企业“零容忍”为目标，强化企业服务办公室的协调、督查、问责职能，完善责权一致的领导分包企业机制，加大明察暗访力度，及时有效处理企业投诉事项，严格追究行政职责过错，让服务企业成为洛阳的“金字招牌”。

改进作风，营造透明高效的政务环境。以洛深合作为契机，大力推进政府学习创新、观念创新、制度创新、管理创新。围绕市委、市政府确定的中心工作，明确责任，限时完成，全面提高政府执行力。大力推进阳光施政，加强对公共资源交易行为的监督管理，实现县（市）公共资源交易中心平台全覆盖，让阳光普照在权力运行的每个角落。加快电子政务建设，实施网上审批，优化审批流程，推动行政审批从“绿色通道”向“高速公路”拓展升级，用政府的高效率带来企业的高效益、赢得群众的好口碑。

勤政廉政，营造风清气正的政治生态环境。持续巩固和深化党的群众路线教育实践活动成果，严防“四风”问题反弹。坚持忠诚履职、谨慎用权，多办顺民意、暖民心的实事、好事，以实际行动取信于民，让群众真心为政府点赞！认真落实中央八项规定精神，严格执行廉洁自律各项规定，切实履行党风廉政建设“一岗双责”。完善惩治和预防腐败体系，加快构建监察、司法、审计联动的行政权力监督机制，切实做到用制度管权管事管人。认真落实财政预算制度，完善公务消费和公务接待制度，严控“三公”经费支出，厉行勤俭节约，反对铺张浪费，用政府的紧日子换来群众的好日子。

各位代表！梦想承载希望，实干开创未来。让我们紧密团结在以习近平同志为总书记的党中央周围，在省委、省政府和市委的坚强领导下，开拓创新、团结奋进、勇于担当、务实重干，为全面完成“十二五”各项目标任务、加快建设名副其实的中原经济区副中心城市、在中原崛起中更出彩而努力奋斗！
